# Modification of sesame (*Sesamum indicum* L.) for Triacylglycerol accumulation in plant biomass for biofuel applications

**DOI:** 10.1016/j.btre.2021.e00668

**Published:** 2021-09-11

**Authors:** C. Muthulakshmi, R. Sivaranjani, S. Selvi

**Affiliations:** Department of Biotechnology, PSG College of Technology, Coimbatore, 641004, Tamil Nadu, India

**Keywords:** Sesame, Triacylglycerol, Biofuel, Transformation, Polymerase chain reaction

## Abstract

Sesame is considered as the queen of oil seeds owing to its high oil content of about 56–62% and good quality oil. Sesame oil alone or in combination with other vegetable oils can yield good quality biodiesel. Sesame biodiesel blends up to 20% yields fuel efficiency and power output on par with mineral diesel but superior in environmental performance. Though biodiesel from edible oil is highly criticized, the demand for renewable energy compels the development of high-performance sesame plants. Triacylglycerol synthesis in flowering plants follows an acyl CoA-dependent and independent manner. This study envisages transgenic approaches to enhance oil production in sesame biomass. The genes of choice for oil enhancement includes *DGAT1, PDAT1, FAD3* and cytochrome b5F. Diacylglycerol acyltransferase (*DGAT*) and phospholipid diacylglycerol acyltransferases (*PDAT*) are key enzymes in TAG synthesis. Fatty acid desaturases (*FAD*) has the ability to enhance specific fatty acids, whereas cytochrome b5 genes augment the process by donating electrons. A combination of the above categories of genes which performed well in terms of oil content in the yeast expression system from our earlier studies is used in Agrobacterium-mediated sesame transformation experiments to evaluate the biodiesel potential of transgenic sesame plants. The transgenic construct with PDAT1 and FAD3 combination yielded a 10% increase in TAG content. The possibility of transgenic sesame as a biodiesel plant is discussed.

## Highlights

• Increased oil biomass in sesame vegetative tissues.
• Enhancement of plant oil biomass plays a chief role in biofuel applications.
• This is a maiden attempt to develop sesame plant for biofuel production.

## 1 Introduction

Vegetable oils play a key role in the agricultural economy as well as industrial applications such as biofuel feedstock. Global demand for vegetable oil consumption is expected to be increased remarkably by 2050 [1, 2]. Depletion of non-renewable resources warrants, production of energy from other sources which are sustainable in nature. Biodiesel is one such energy obtained from plant or algal oil that can be cultivated and harvested successively that serves as a substitute for fossil fuel [3,4].Biodiesel is an imperative category of biofuel derived from animal fat, vegetable oil, fatty acid produced from yeast, bacteria, and microalgae which is leading importance worldwide [5]. Oil rich plant products such as seeds, fruits; nuts and bran are extensively used as feedstock to meet the growing demand on fossil fuel. There are several edible oilseeds including soybean (*Glycine max*), sunflower (*Helianthus annus*), peanut (*Arachis hypogaea*), oil palm (*Elaeis guineensis*), sesame (*Sesamum indicum*) and non-edible vegetable oil such as *Jatropha curcas*, rapeseed (*Brassica napus*) are the existing vegetable oil production sources [1]. Metabolic Engineering strategies to produce copious amount of oil from plants will turn out to be a cost-effective method for biodiesel production [6]. Genetic engineering of lipid biosynthesis pathway genes in plants promises an economical and feasible method of surplus oil production. Several pieces of evidence have been made through the metabolic engineering of lipid biosynthesis genes [7]. Triacylglycerol (TAG) is the main form of storage lipid accumulated in plant oil. TAG biosynthesis mainly takes place in two compartments plastid and endoplasmic reticulum. *DGAT* (AcylCoA: diacylglycerol acyltransferase) and *PDAT* (phospholipid diacylglycerol acyltransferase) are two main enzymes involved in TAG biosynthesis. They are rate-limiting enzymes that act in the final step of TAG synthesis [8]. Fatty acid desaturases are other most important enzymes which are involved in polyunsaturated fatty acid biosynthesis [9].

In plants, fatty acid synthesis takes place in two different pathways such as prokaryotic (chloroplast) and eukaryotic (endoplasmic reticulum) and these pathways are encoded by set of genes [10].Nuclear genes code for fatty acid desaturases, which differ in substrate specificity and subcellular location. They are essential for the appropriate formation and function of biological membranes [11] and metabolic channeling [12]. The *FAD3* gene encodes omega-3 desaturases, which catalyses the conversion of linoleic acid to *α*-linolenic acid (ALA) [13]. Overexpression of the ω−3 fatty acid desaturases Bn*FAD3* from *Brassica napus* and St*FAD7* from *Solanum tuberosum* in tomato increased cold stress resistance and changed fatty acid composition in leaves and fruits, with an increase in the 18:3/18:2 ratio [14]. *Insilico* comparative genomic analyses by Shar[15]ma and Chauhan (2012) had indicated that variations in *FAD2, FAD3*, Stearoyl desaturase, *DGAT-1*, and *DGAT-2* will be beneficial in increasing plant oil content. Cytochrome b5 (Cb5) is a heme-binding protein that is located with the endoplasmic reticulum and outer membrane of mitochondria. In plastids, reduced ferredoxin offers electrons to desaturases, while in endoplasmic reticulum Cb5 gives electrons to both FAD2 and *FAD3* [16]. Recently, genetic engineering approaches were applied to improve biofuel feedstock production from the plant source. Fatty acid composition is the key for biodiesel properties. Cost effective and good quality biodiesel can be achieved by suitably modifying the chain length and saturation levels of fatty acids [17].Cold flow properties and viscosity of biodiesel is solely dependent on chain length of fatty acids. Various studies have established that specific acyl-ACP thioesterases play a crucial role in cleaving fatty acids from growing acyl-ACP in lipid metabolism. Resulting in 90% of short and medium-chain fatty acids observed in *Umbellularia californica* and *Cuphea hookeriana* seeds [18]. Another important aspect to be considered from a plant source as a biofuel is the ‘concept of food versus fuel’. Presently, major biofuel plants consist of food crops such as soybean, peanut, olive, and rapeseed. Next to food crops, *Jatropha curcas* a biodiesel crop which yields 1.5–2.5 T-biodiesel/ha [19]. In rapeseed, expression of Acetyl-CoA Carboxylase (ACCase) enzyme targeted towards chloroplast has achieved a 5% increase in oil content of seeds [20]. Increasing the seed oil and seed weight in Indian mustard (*Brassica juncea*) was achieved by overexpression of the Arabidopsis At*DGAT1* gene. The gene was mobilized into mustard through Agrobacterium-mediated transformation. An increased seed oil content of 8.3% was observed in transgenic mustard than the wild type plants [21]. Other than plants; microalgae play a major role in bioenergy application in industries. Microalgal biomass is a natural source for various applications such as carbon fixation, genetic manipulation of enhanced product yield and recovery for biofuel industry [22]. A naturally occurring ethanologen is *Zymomonas mobilis*, a gram-negative bacterium. It possesses a number of useful industrial biocatalyst features, including high ethanol productivity and tolerance. Z. mobilis consumes glucose for ethanol production faster than *Saccharomyces cerevisiae* due to its wide cell surface area, resulting in better ethanol output [23]. As model organisms for bio alcohol synthesis, yeasts have long attracted the scientific community's attention. Higher amounts of saturated and monounsaturated fatty acids may be regarded ideal for fuel quality in *Saccharomyces cerevisiae*. Unexplored biological variety in microorganisms such as yeasts should be used for bioenergy–bio refinery-based applications, comparable to microalgae [24].

This study aims to investigate effective candidate genes for biofuel production in the sesame plant which already has high oil content in seed. Sesame (*Sesamum indicum* L.) is considered as a traditional oil crop with high levels of oleic and linoleic fatty acids. The main goal of this study is to develop sesame transgenic plants with high oil content. Fatty acid desaturase 3 expression in seeds is meager compared to vegetative tissues. *FAD3* in combination with the other three genes would enhance the oil in vegetative tissues. Hence recombinant constructs were developed for constitutive expression of *DGAT1, PDAT1,* and Cytochrome b5 genes with *FAD3* genes. In plants, triacylglycerol can be accumulated in high levels in oilseeds which support seed germination and development. However, TAG content in non-seed tissues is very low; it ranges from 0.04% to 0.2% of the dry weight in Arabidopsis leaf tissues [25]. Keeping this in mind increase in TAG accumulation in plant vegetative tissues employing genetic engineering approaches will enable the generation of high oil content in plant biomass.

## 2 Materials and methods

### 2.1 Plant materials

Sesame plants were grown in MS (Murashige and Skoog) basal medium using TMV7 variety. The conditions used for growth at 25± 2 °C temperature with a photoperiod of 16 h cool-white light and 8 h dark [26]. Sesame tissues of various developmental stages were collected and RNA stabilization reagent (Qiagen, USA) was used to store them at -80 °C.

### 2.2 *Insilico***gene analyses**

Sesame *DGAT1, PDAT1, FAD3* and Cytochrome b5 genes from the database were retrieved using BLASTN search using Arabidopsis sequences as queries against assembled genome of *S.indicum*. PCR Primers were designed using the retrieved sequences. Gene specific PCR amplification primers were selected using the Primer 3 program for *DGAT1, PDAT1, FAD3* and Cytochrome b5were listed in Supplementary Table 1 and 2.

### 2.3 Protein sequence, structure, and phylogenetic analysis

A comparative sequence analysis was performed on sesame protein responsible for TAG accumulation. Homologous sequences from various plants were retrieved from NCBI and the list is provided in the Supplementary file:1. Sequences were aligned by MUSCLE (MEGA7) software and phylogenetic tree was constructed by Maximum-likelihood (ML) method. ExPASy (http://www.cn.expasy.org/tools) tools were used to analyze the molecular weights (MWs) and isoelectric points (pIs) of the protein selected. Transient signal peptides were predicted using TargetP1.1 (http://www.cbs.dtu.dk/services/TargetP/). Conserved domain detection was performed using SMART (http://smart.embl-heidelberg.de/). Trans membrane helices of *DGAT1, PDAT1, FAD3*, and Cyt b5-F protein sequence were predicted using TMHMM Server v. 2.0 (http://www.cbs.dtu.dk/services/TMHMM/). Genomic structures of sesame *DGAT1, PDAT1, FAD3*, and Cb5 genes were predicted using Gene structure display server 2.0 (http://gsds.cbi.pku.edu.cn/).

### 2.4 **Isolation of full-length sesame *DGAT1, PDAT1, FAD3*, and cytochrome b5 genes**

Total RNA were extracted from leaves, stems, roots, flowers, developing seed and mature seed using RNeasy plant mini kit (Qiagen, USA). RNA purity and integrity were observed by agarose gel electrophoresis and first-strand cDNA was achieved using the Revert Aid First Strand cDNA synthesis kit (Thermo Scientific, USA). PCR amplification of full length *DGAT1, PDAT1, FAD3* and Cyt b5 genes was performed using Ex Taq DNA polymerase (Takara, Japan) with the following conditions, denaturation at 98 °C for 10 s, annealing at 60 °C for 30 s, and extension at 72 °C for 1 min/Kb. The amplicons were resolved in 1% agarose gel and purified using Gel extraction kit (Thermo Scientific, USA) then cloned into pTZ57R/T vector (Thermo Scientific, USA).The restriction enzymes was underlined and mentioned in Supplementary Table1.

### 2.5 Quantitative real-time PCR

Total RNA from various sesame tissues was used for qPCR experiments. One microgram of purified RNA was taken for first strand cDNA synthesis. Gene-specific primers were designed for *FAD3*, Cb5 genes and ubiquitin (UBQ 6) was used as a housekeeping gene. Quantitative RT-PCR was performed using SYBR Premix Ex Taq II (TliRNase H Plus) (Takara, Japan) according to manufacturer's instructions. Each 10 µl reaction comprised of 100 ng of template, 5 µl of SYBR Premix, and 0.2 µl (200 nM) of each primer. CFX96 Real-Time PCR detection system (Bio-Rad, USA) was used for quantitative RT-PCR experiment. The PCR amplification condition: 95 °C for 30 s and 40 cycles of 95 °C for 5 s and 60 °C for 30 s followed by 95 °C for 15 s, 60 °C for 1 min, 95 °C for 15 s (melt curve). All reactions were performed for three independent replicates and relative gene expression was calculated using 2^−∆∆Ct^ method. Total RNA were isolated from transformed and non-transformed sesame plants for RT-PCR analysis. The PCR products were electrophoresed on 1.5% agarose gel and visualized on Gel documentation system (Alpha imager). Relative gene expression levels of NPTII gene were analysed using 2^−∆∆Ct^ method.

### 2.6 Recombinant gene constructs

The expression vector which harboured any one of the full length *DGAT1*/*PDAT1*/Cb5 gene was combined with *FAD3* gene, and a kanamycin resistance gene and each of which was driven by endogenous CaMV 35S promoter and NOS terminator. The *DGAT1*/*PDAT1*/Cb5 genes were inserted into the XbaI and XmaI sites while the *FAD3* gene was inserted into XmaI and SnaBI sites of pBI121 vector. All three gene constructs were transformed into *Agrobacterium tumefaciens* LBA 4404 strain using Freeze-thaw method [27].Schematic representation of binary vector pBI121 harbouring the genes of interest were shown in supplementary file 2.

### 2.7 Plant materials and growth conditions

Seeds of TMV-7 sesame variety were used in transformation experiments. Seeds were obtained from Oilseed Research Station, Tindivanam affiliated to Tamil Nadu Agricultural University, Coimbatore. Sesame seeds were surface sterilized with 70% ethanol for 1 min 30 s, then with 0.1% (w/v) HgCl_2_ for 4 min, thoroughly washed 4–5 times with sterile water placed in petriplates containing half MS (Murashige and Skoog) (Himedia, India) basal medium for growth and maintained at 25±2 °C with 16 h cool-white fluorescent lights and 8 h dark. For direct organogenesis, the cotyledonary explants were dissected from the seeds and the embryonic axis was removed completely. The de-embryonated cotyledons were cultured on pre-regeneration medium containing 29 μM BAP + 8 μM IAA + 29 μM AgNO 3 + MS (6% sucrose) were maintained for one week. We have optimised hormone concentration, pre-culture period, infection time, co-cultivation period and kanamycin concentration (Unpublished data). The transformation efficiency for hormone concentration was reached 47.5%; frequency of explants survived in pre-culture period was attained at 4 days, frequency of explants survived in co-cultivation period for 3 days and kanamycin concentration 50 mg/l was used and reached the transformation efficiency of 23.1%.

### 2.8 Agrobacterium mediated transformation and shoot regeneration

Transformation in sesame explants were carried using transformed *Agrobacterium tumefaciens* single colony having the desired gene combination in the binary pBI121 vector. The antibiotics used in selection were kanamycin and rifampicin and the growth conditions was 200 rpm at 28 °C for 12 h. *A.tumefaciens* liquid culture was centrifuged at 4000× *g* for 10 min to pellet the cells. The cells were suspended in 50 ml of liquid MS medium and 20 µl of 20 μM acetosyringone (Murashige and Skoog 1962). The de-embryonated cotyledons were incubated in the liquid agrobacterium for 15 min and dried on sterile filter paper. Infected explants were then incubated in dark for 3 days on co-cultivation medium (pre regeneration medium + 20 μM acetosyringone). Following co-cultivation, explants were washed two times with sterile water supplemented with 500 mg/l cefotaxime (Himedia, India) and placed in pre culture media containing cefotaxime (500 mg/l) and 50 mg/l kanamycin. Explants were sub cultured with two week intervals and transferred to shoot elongation medium containing MS basal medium with 0.3 mg/l GA_3_.

### 2.9 Rooting

The elongated shoots were transferred to the MS medium containing IAA 4 µM/L with 5 mg/L kanamycin. Transformed plants were moved to pots containing autoclaved mixture of coir peat: vermiculite: perlite (1:1:1) for hardening.

### 2.10 Histochemical staining of *β-glucuronidase* activity

Samples were collected from randomly selected putative transformants and they were examined for GUS activity using histochemical staining [28]. Samples were incubated in freshly prepared 5‑bromo-4‑chloro-3-indolyl-*β*-glucuronidase (X-gluc) (Duchefa Biochemie, Netherlands) substrate solution at 37 °C for 16 h in dark. This was followed by washing with decolorizing solution which contains methanol and acetone in the ratio of 3:1.

### 2.11 Sesame genomic DNA extraction

DNA was extracted from transformed and non-transformed control sesame plants by modified CTAB method [29]. One gram of fresh leaves were ground into fine powder using liquid nitrogen then 500 µl of grinding buffer (100 mM Tris-Hcl,5 mM EDTA,0.35 M sorbitol and 2% PVP) was added. The contents were centrifuged for 10 min at 6500 rpm. The supernatant was discarded and 500 µl of lysis buffer (100 mM Tris-Hcl, 20 mM EDTA, 1.42 M NaCl, 2% CTAB, 2%PVP, 5 mM ascorbic acid and 4 mM diethyldithiocarbamic acid) was added. The above lysate was incubated at 65 °C for 30 min. Chloroform: isoamylalcohol (24:1) extraction was performed and the aqueous phase was transferred to new tube. DNA was precipitated using one volume of ice cold isopropanol and incubation at −20 °C for 30 min. Pellet was collected by centrifugation at 12,000 rpm for 10 min and washed with 70% ethanol. DNA was dissolved in TE buffer and treated with RNase and the DNA was assessed by 0.8% agarose gel.

### 2.12 PCR analysis of putative transformed shoots

Putative transformed shoots were screened for presence of *Npt II* gene by PCR. The 790 bp sequence of *Npt II* gene was amplified using gene specific primers listed in Table 1. Each PCR reaction was done in 20 µl reaction consisting of 2 µl of 10× reaction buffer, 100 ng genomic DNA, 10 mM dNTPs, 10 µM of each primer, and 1 U of Taq polymerase (Genet bio, Korea).The amplification reaction was performed in a thermal cycler (Agilent Technologies, USA) under the following conditions: initial denaturation 95 °C for 5 min, then 35 cycles of denaturation 95 °C for 30 s, annealing 56 °C and 60 °C for 30 s and elongation 72 °C for 1 min and final elongation 72 °C for 10 min. The amplified product was electrophoresed on 1% agarose gel and photographed through gel documentation system (Alpha imager).

### 2.13 Lipid extraction

Total lipids were extracted from transformed and non-transformed leaf tissues of sesame [30]. Two gram of leaf tissues was taken and then 6 ml of preheated isopropanol was added. Then the samples were incubated at 75 °C for 15 min. The samples were chilled to room temperature and then 3 ml of chloroform and 1.2 ml of water was added. The samples were incubated with occasional shaking for 1 hour and separated by 3000 rpm for 5 min. The lipid extract was transferred to a new tube then extracted with 8 ml of chloroform: methanol (2:1v/v). Combined the lipid extracts were added with 2 ml of 1 M KCl. The samples were mixed and then centrifuged at 3000 rpm for 5 min and then discarded the upper phase. To the lower phase 4 ml of water was added, centrifuged and the upper phase was discarded. The samples were dried under nitrogen stream. Lipids were trans esterified with 2.5% of H_2_So_4_ in methanol [31]. Thin layer chromatography (TLC) was used to separate TAGs from total lipids. The solvent used were hexane/diethyl ether/acetic acid in 80:20:1 ratio. Silica plates were immersed in 10% copper sulfate pentahydrate solution to visualize the lipid spot. Visualization of lipid spot in TLC is performed by the procedure given [32].

### 2.14 Total oil content calculation

Fatty acid methyl esters (FAME) were calculated from the lipid weight and the molecular weight [33]. The oil content was measured by comparing the concentration of fatty acids using the peak area. Oil content was calculated using the formula: percent oil by weight = 100 × ((4 x total mol FAME/3) + total g FAME)/g tissue, where 4 is the Mr difference between TAG and three moles of FAME. The data represented was on dried weight basis (DW) and the average of three independent replicates.

## 3 Results

### 3.1 *Insilico* identification and characterization of selected sesame oil augmenting genes

The annotation of sesame genome furnishes the information for categorizing genes involved in TAG synthesis. We have shortlisted one *DGAT1*, one *PDAT1*, one *FAD3* and one Cyt b5 genes in different chromosomal locations of sesame genome. The evolutionary relationship of these genes was examined by constructing a phylogenetic tree by maximum likelihood method (Supplementary file.1).The segregation of *DGAT* and PDAT family of proteins into two separate clades were observed. All four isoforms of sesame *PDAT1* genes are clustered together with *Erythranthe guttata*. Three isoforms of sesame *DGAT1*-A1, *DGAT1*-A2 and *DGAT1*-B1 are clustered together; other two isoforms Si*DGAT1*, Si*DGAT1*-B2 were paired with *E.guttata*. Phylogenetic relationship of omega 3 fatty acid desaturase *FAD3* gene was clustered with *Perilla fructens FAD3*. Six isoforms of sesame Cyt b5 isoforms were clustered with *E.guttata*. SiCyt b5-C isoform was clustered with *Vernicia fordii* and SiCyt b5-D isoform was clustered with *Glycine* max Cyt b5 gene. Protein characteristics of *FAD3* and Cyt b5-F genes were stated in Supplementary Table.3. Subcellular localization of the proteins was predicted by TargetP1.1 program and conserved domains in the genes were analysed by SMART program were shown in Supplementary Table 4. Gene Structure Display Server (GSDS) is employed in predicting the structure of these genes shown in Supplementary Figure1.

### 3.2 Differential mRNA expression of sesame *DGAT1, PDAT1, FAD3* and Cyt b5-F genes

**Fig. 1:**
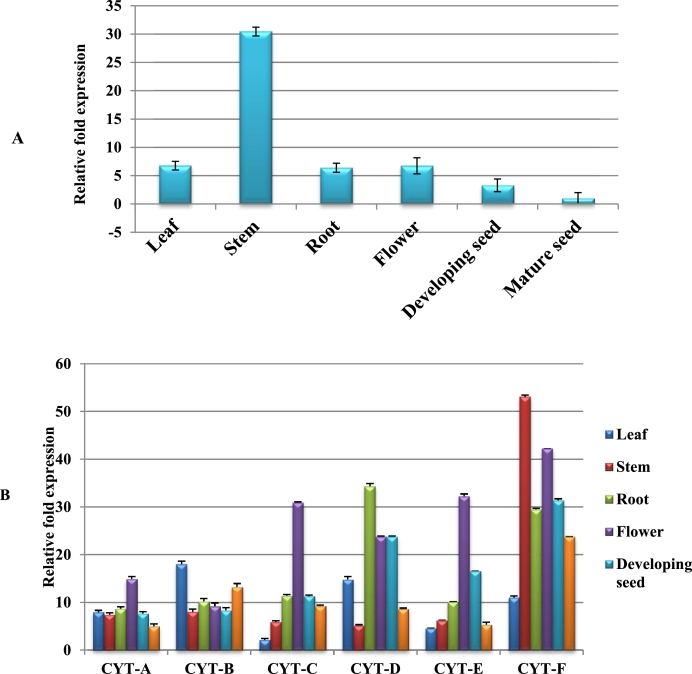
Expression analysis of omega 3 desaturase genes FAD3 (A) and Cyt b5 (B) genes in sesame using Quantitative RT-PCR. Developing tissues- leaf, stem, root, flower developing seed and mature seed. The mRNA abundance was normalized with respect to ubiquitin 6 gene as an endogenous control. The bars represent the standard deviation of three biological replicates. .

The role of sesame *DGAT1, PDAT1, FAD3* and Cyt b5-F genes can be understood by analysing their expression pattern in different stages of crop growth and various tissues in plant development. The relative expression is compared with a constitutively expressed ubiquitin gene (UBQ 6). Si*DGAT1* gene recorded the highest transcript accumulation at mature seeds compared to other tissues tested whereas Si*PDAT1* transcript was detected preferentially in flowers than other tissues in our earlier experiments. Expression pattern of omega 3 fatty acid desaturase in microsomal (*FAD3*) were analysed in different tissues and its expression was higher in stem tissues (Fig. 1A). In this study, we preferred microsomal *FAD3* for co-expression with *DGAT1* and *PDAT1* in *Agrobacterium* mediated transformation. On the other hand, six Cyt b5 isoforms were selected and their expression were analysed. Cyt b5-F showed higher expression in most of the tissues examined (Fig. 1B).

### 3.3 Transformation and shoot regeneration through direct organogenesis

**Fig. 2:**
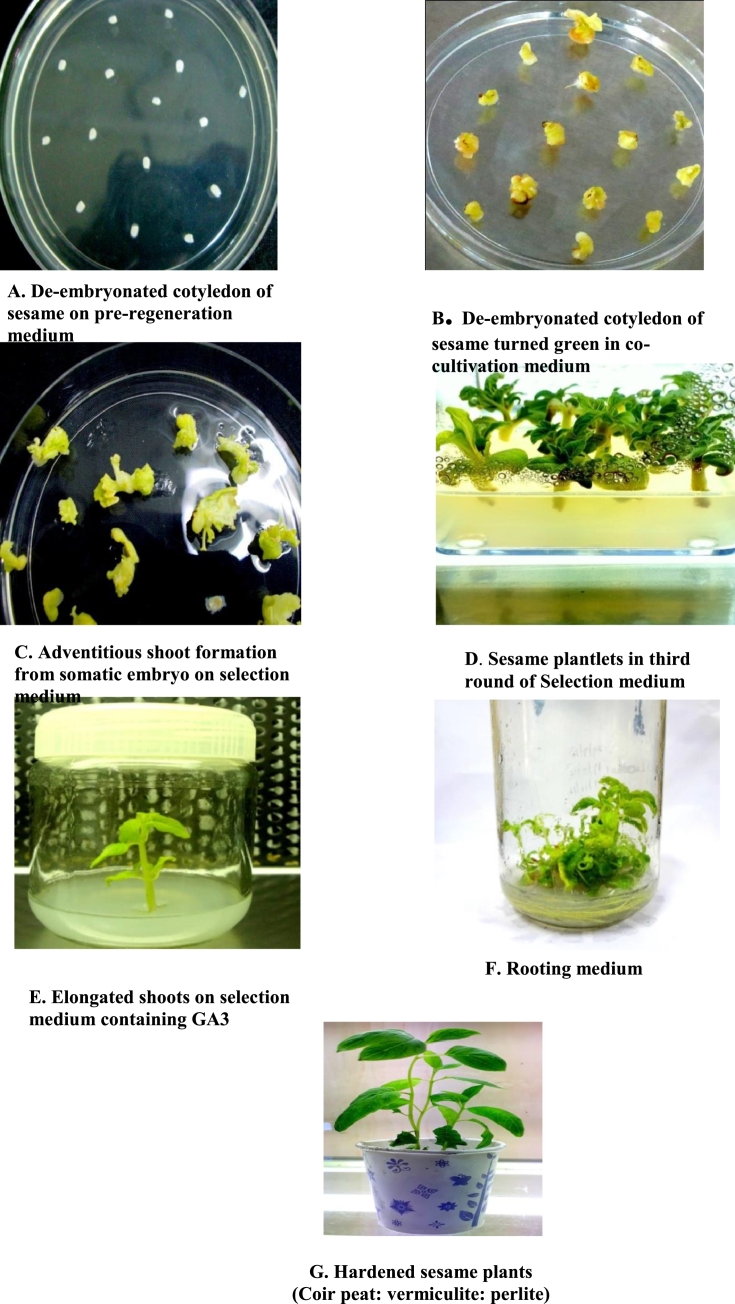
Stages of shoot and root development of transformed sesame plants.

The sesame variety preferred for our study was TMV7 on the basis of high yield of about 820 kg/ha, tolerant to root rot disease and suitable for value addition in comparison with other local cultivars. The embryos were excised and de-embryonated cotyledons were used as explants for Agrobacterium-mediated transformation (Fig. 2A). After four days of incubation at 25 ± 1 °C, these explants turned green and they were used for transformation (Fig. 2B). De-embryonated cotyledons were then infected with freshly grown culture of *A.tumefaciens* carrying the binary vector pBI121.Co-cultivation period, infection time and acetosyringone concentration in the co-cultivation medium were standardized based on the transformation efficiency of the explants (Unpublished data). Following co-cultivation, de-embryonated cotyledons were washed with cefotaxime (500 mg/l) and placed in a medium containing cefotaxime (500 mg/l) and kanamycin (50 mg/l). Three rounds of selection were performed in ten days interval, during this period somatic embryos showed adventitious shoot formation (Fig. 2C and D).They were transferred to MS medium containing 0.3 mg/l GA_3_ for shoot elongation (Fig. 2E). The elongated shoots were then carefully removed and placed in a medium containing 4 µM IAA and 5 mg/l kanamycin for rooting (Fig. 2F). After formation of roots the plants were transferred to hardening media with ratio of 1:1:1 pith: vermiculite: perlite respectively (Fig. 2G).

### 3.4 P**CR analysis of *NPTII* gene for transformants**

Genomic DNA isolated from leaves of twelve putative transformants of sesame (*S. indicum* L.) and one control (wild type) plant (uninfected with *A. tumefaciens*) was used for presence of marker gene by PCR analysis (Supplementary Figure.4).

### 3.5 Quantitative RT-PCR analysis of *NPTII* gene in transgenic lines of sesame

**Fig. 3:**
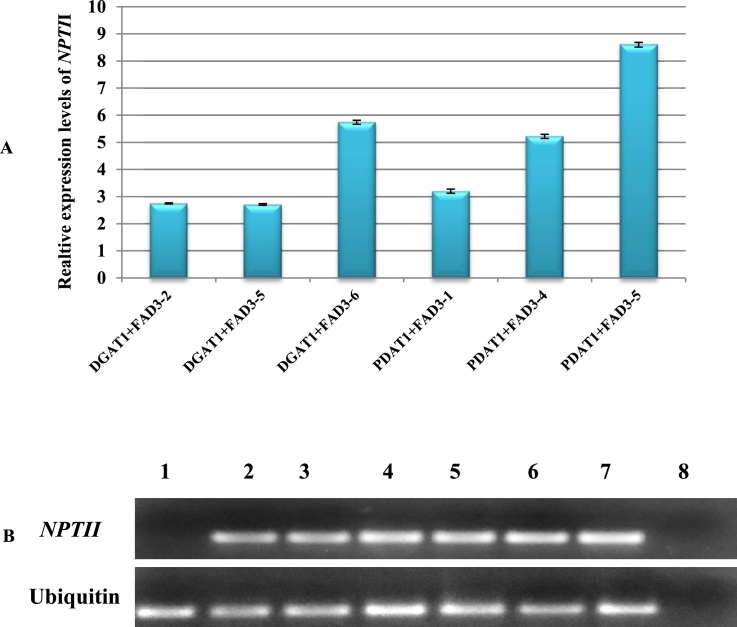
**Quantitative RT-PCR confirmation of *NPTII* gene in transformed sesame lines.** A. Relative expression levels of *NPTII* gene in transformed sesame lines when compared to untransformed control. Ubiquitin 6 gene was used as an endogenous control. The bars represent the standard deviation of three independent replicates. B. Agarose gel electrophoresis of qRT-PCR amplification products of *NPTII* and Ubiquitin gene. Lane 1: Untransformed sesame plants, lane 2–4: transformed sesame plants having DGAT1 + FAD3 gene, lane 5–7: transformed sesame plants having PDAT1+FAD3 gene and lane 8: no template control (NTC).

The stable integration of transgene in sesame transgenic lines was confirmed using RT-PCR analysis. For RT-PCR analysis, three transformed lines of *DGAT1* + *FAD3* and *PDAT1* + *FAD3* were chosen. The mRNA expression analysis of *NPTII* gene showed higher levels in *PDAT1* + *FAD3*–5 transformed line when compared to other transformed lines (Fig. 3A). All six lines showed the presence of *NPTII* gene expression. In control plants, only ubiquitin gene showed the expression and thus indicating the absence of *NPTII* gene in transformed tissues (Fig. 3B).

### 3.6 Expression of reporter gene in transformants

**Fig. 4:**
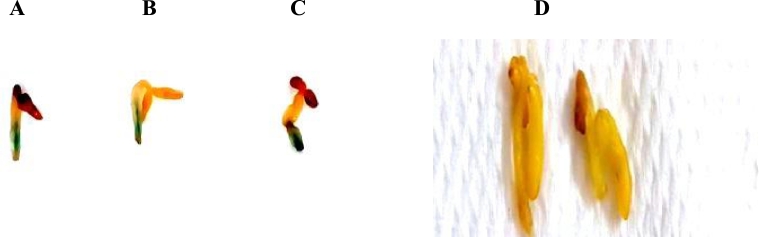
**GUS assay for putative transformed sesame plants.** A. DGAT1 + FAD3 B.PDAT1 + FAD3 C. Cyt b5-*F* + FAD3 D. Untransformed sesame plant (Control).

The putatively transformed tissues of eight to ten weeks old were randomly selected from selection medium and were subjected to GUS expression analysis. The expression of GUS gene in explants that survived after two rounds of selection was visualized (Fig. 4A–D).

### 3.7 Analysis of TAG accumulation in sesame transformed leaves

**Fig. 5:**
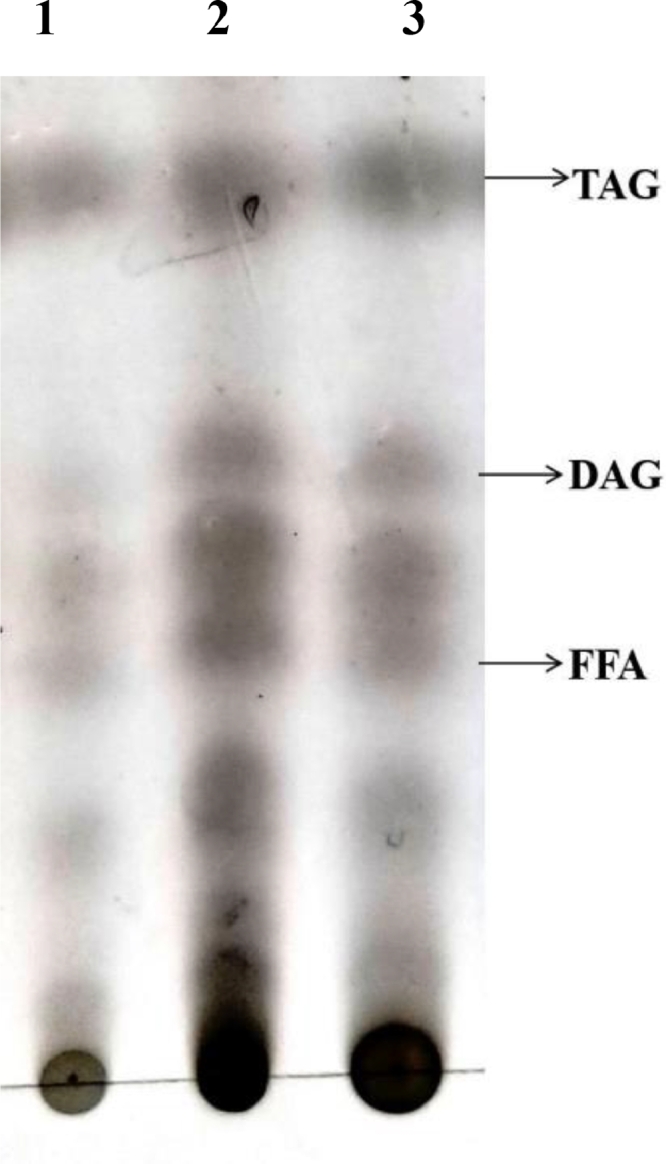
**A: TLC separation of total lipids isolated from sesame leaves.** 1. Untransformed sesame plant (control), 2.DGAT1 + FAD3 and 3.PDAT1 + FAD3. **B: TAG accumulation in sesame leaf tissues.** Leaf TAG content is shown for control (Untransformed plant), sesame plants transformed with DGAT1 + FAD3 and sesame plants transformed with PDAT1 + FAD3. The bars represent the standard deviation of three technical replicates.

Total lipids were extracted from transformed and non-transformed leaves of sesame for analysis of TAG accumulation. In order to verify the TAG from total lipids, TLC separation was performed (Fig. 5A). The levels of TAG were compared. The accumulation of TAG particularly in leaf biomass was achieved only through constitutive promoter in wild type and transformed plants. The pBI121 constitutive promoter containing the *DGAT1* and *FAD3* gene combination recorded TAG accumulation of about 24.2 (% Dried weight DW) whereas the sesame plant transformed with *PDAT1* and *FAD3* genes showed 26.7 (%Dried weight DW) TAG content than non-transformed plants with only 15.2% DW (Fig. 5B).

## 4 Discussion

*Sesamum indicum* is a prominent oil seed crop, which can accumulate high levels of polyunsaturated fatty acids and serve as one of the chief sources of health-boosting vegetable oil and other plant based by-products**.** The main objective of this study is to identify the candidates involved in TAG synthesis in plant biomass of sesame. In our earlier study, we identified six *DGAT1* isoforms, *DGAT*2, four *PDAT1* isoforms and *PDAT2* genes in sesame genome [34]. Present *insilico* characterization revealed that the members of *DGAT1* and *PDAT1* have several features conserved in all plants at both the gene and protein levels which were also observed by Pan et [35]al. (2015). The presence of MBOAT domain and nine other transmembrane domains in sesame *DGAT1* suggest that it is a membrane protein and it is also localized in the ER (Supplementary Table.3). Similarly, groundnut (*Arachis hypogaea*) *DGAT1* has MBOAT region and nine transmembrane domains it was highly conserved among other *DGAT1* [36]. The existence of LCAT like domain shows that it belongs to LCAT superfamily and these *PDAT1* are membrane proteins which contains single transmembrane domain and localized in plasma membrane. Similarly, in *Myrmecia incisa* PDAT protein was located in the plasma membrane whereas other PDAT from *Camelina sativa* was located in endoplasmic reticulum [37, 38]. Omega 3 fatty acid desaturase *FAD3* protein from sesame contains an uncharacterised DUF3474 domain and FA desaturase domain. Three transmembrane domains were predicted in sesame *FAD3* protein and it was located in endoplasmic reticulum. Similarly in *Perilla fructens FAD3* the same domains were present which was highly conserved among eukaryotes and two transmembrane domains were predicted [39]. The three genes chosen for this study have transmembrane domains but different localization pattern suggestive of expression in diverse plant parts. Although for enhancement of TAG accumulation which includes metabolic flux improvement, biosynthetic genes in fatty acid metabolism, increasing energy and carbon intake, overexpression of lipid metabolism genes such as Accase, *DGAT,PDAT*, KAS III, fatty acid synthase, ACP thioesterase these are some of the targeted genes involved in genetic engineering aspects [40,41].

Expression profile of *DGAT1, PDAT1, FAD3* and Cyt b5-F genes suggests that these genes are regulated in a tissue and stage specific manner (Fig. 3). Sesame *DGAT1* predominantly expressed in mature seeds [34] whereas in *Arachis hypogaea DGAT1*–2 isoform shows higher expression in seed tissues [36]. The expression pattern of sesame *PDAT1* gene was higher in flower tissues [34] than other tissues examined. Similarly, in *Camelina sativa PDAT1*-C was highly expressed in leaf and flower tissues [38]. The above observation suggests that *PDAT1* is a better candidate than *DGAT* in TAG accumulation in biomass than seeds. Our result also is in concordance with this (Fig 5A).The transcript abundance of *FAD3* gene was higher in stem tissues than other tissues (Fig. 1A).Similarly, in *Salvia hispanica FAD3* showed high expression stem and also in early seed tissues [39]. The expression profile of six cytochrome b5 isoforms of sesame were analysed in various tissues (Fig. 1B). The transcript abundance was higher in stem tissues of Cyt -F isoform, moreover in Cyt-C, Cyt-D, Cyt-E and Cyt-F also displayed higher in flower tissues. Similarly, in *Glycine* max cytochrome b5 isoforms revealed that root, leaf, flower and seed tissues have constitutive expression pattern [16]. In sesame *FAD3* which expresses in non-seed parts is chosen and the constitutively expressed cytochrome b5 will also be a better choice. The present transgenic experiment is directed for TAG accumulation throughout the plant biomass for biodiesel applications. Plant lipid metabolism is regulated by various biochemical pathways and it contains different rate-limiting enzymes [42]. Triacylglycerol are not constitutively expressed in high quantities due to regulation of these key enzymes. Overexpression of single rate-limiting genes shown only significant amount of oil accumulation in plant tissues [43]. Moreover, simultaneous insertion of multiple genes it would give increased accumulation of TAG in plant biomass. Overexpression of *DGAT1*, OLEOSIN1 and WRI1 genes enhanced the TAG content in tobacco leaf to 15% of DW was achieved through simultaneous multiple gene targets [44].

To increase the oil content in vegetative tissues, we overexpressed the sesame *DGAT1, PDAT1* and *FAD3* genes by inserting an additional copy to the existing native genes. In order to get high TAG synthesis in biomass, the expression of *DGAT1* and *PDAT1* with *FAD3* ORF was driven by CaMV 35S promoter. Total lipids were extracted from transformed and non-transformed sesame plants and separated by TLC (Fig. 5A). Expression of DAGT1 and *PDAT1* with *FAD3* genes led to noteworthy improvement in oil content in the leaf (Fig. 5B). The accumulation of TAG was higher 26.7(%DW) in *PDAT1* + *FAD3* combined construct compared to the untransformed wild type 15.2 (%DW). However, significant expression of three fold increase in TAG content was observed in *Camelina sativa* [38]. This study is a maiden attempt to modify sesame plant for biodiesel applications. In future, inclusion of cytochrome b5 in transgenic studies to meet the electron demand during fatty acid synthesis might yield a better TAG accumulation throughout the plant.

## 5 Conclusion

Vegetable oil consumption is increased globally and it is also used for several non-edible purposes like biofuel production, pharmaceutical companies etc. Enhancement of oil yield through conventional breeding methodologies is still limited due lack of genetic variability and resources. To address this problem the study on lipid biosynthesis pathways play a major role through genetic modification approaches. Selection of promoters and target gene plays an imperative criterion to establishing the industrially feasible biomass production platform.

## Declaration of Competing Interest

The authors declare that no conflict of interest.

## Appendix Supplementary materials

Image, application 1

Image, application 2

Image, application 3

## References

[1] Harwood J.L., Ramli U.S., Tang M., Quant P.A., Weselake R.J., Fawcett T., Guschina I.A. (2013). Regulation and enhancement of lipid accumulation in oil crops: the use of metabolic control analysis for informed genetic manipulation. Eur. J. Lipid Sci. Tech..

[2] Samarth N.B., Mahanwar P.A. (2015). Modified vegetable oil based additives as a future polymeric material. Open J. Org. Polym. Mater..

[3] Demirbas A. (2009). Progress and recent trends in biodiesel fuels. Energy Convers. Manag..

[4] López B.C., Cerdán L.E., Medina A.R., López E.N., Valverde L.M., Peña E.H., Grima E.M. (2015). Production of biodiesel from vegetable oil and microalgae by fatty acid extraction and enzymatic esterification. J. Biosci. Bioeng..

[5] Kalscheuer R., Stöveken T., Steinbüchel A. (2007). Engineered microorganisms for sustainable production of diesel fuel and other oleochemicals from renewable plant biomass. Int. Sugar J..

[6] Blatti J.L., Michaud J., Burkart M.D. (2013). Engineering fatty acid biosynthesis in microalgae for sustainable biodiesel. Curr. Opin. Chem. Biol..

[7] Yu W.L., Ansari W., Schoepp N.G., Hannon M.J., Mayfield S.P., Burkart M.D. (2011). Modifications of the metabolic pathways of lipid and triacylglycerol production in microalgae. Microb. Cell Fact..

[8] Turchetto-Zolet A.C., Maraschin F.S., de Morais G.L., Cagliari A., Andrade C.M., Margis-Pinheiro M., Margis R. (2011). Evolutionary view of acyl-CoA diacylglycerol acyltransferase (DGAT), a key enzyme in neutral lipid biosynthesis. BMC Evol. Biol..

[9] Zäuner S., Jochum W., Bigorowski T., Benning C. (2012). A cytochrome b5-containing plastid-located fatty acid desaturase from Chlamydomonas reinhardtii. Eukaryot. Cell.

[10] Somerville C., Browse J. (1991). Plant lipids: metabolism, mutants, and membranes.. Science.

[11] Chen L., Wang L., Wang H., Sun R., You L., Zheng Y., Yuan Y., Li D. (2012). Identification and characterization of a plastidial ω-3 fatty acid desaturase EgFAD8 from oil palm (Elaeis guineensis Jacq.) and its promoter response to light and low temperature. PLoS ONE.

[12] Lou Y., Schwender J., Shanklin J. (2014). FAD2 and FAD3 desaturases form heterodimers that facilitate metabolic channeling in vivo. J. Biol. Chem..

[14] Yadav N.S., Wierzbicki A., Aegerter M., Caster C.S., Pérez-Grau L., Kinney A.J., Hitz W.D., Booth J.R., Schweiger B., Stecca K.L. (1993). Cloning of higher plant omega-3 fatty acid desaturases. Plant Physiol..

[13] Domínguez T., Hernández M.L., Pennycooke J.C., Jiménez P., Martínez-Rivas J.M., Sanz C., Stockinger E.J., Sánchez-Serrano J.J., Sanmartín M. (2010). Increasing ω-3 desaturase expression in tomato results in altered aroma profile and enhanced resistance to cold stress. Plant Physiol..

[15] Sharma A., Chauhan R.S. (2012). In silico identification and comparative genomics of candidate genes involved in biosynthesis and accumulation of seed oil in plants. Comp. Funct. Genomics.

[16] Kumar R., Tran L.S.P., Neelakandan A.K., Nguyen H.T. (2012). Higher plant cytochrome b5 polypeptides modulate fatty acid desaturation. PLoS ONE.

[17] Knothe G., Krahl J., Van Gerpen J. (2005).

[18] Davies H.M. (1993). Medium chain acyl-ACP hydrolysis activities of developing oilseeds. Phytochemistry.

[19] Murphy (2012) http://echa.europa.eu/documents/10162/4410566/the_status_of_industrial_vegetable_oils_from_genetically_modified_plants_expert_report_en.pdf. Accessed 11 Feb 2013</bib>.

[20] Roesler K., Shintani D., Savage L., Boddupalli S., Ohlrogge J. (1997). Targeting of the Arabidopsis homomeric acetyl-coenzyme A carboxylase to plastids of rapeseeds. Plant Physiol..

[21] Savadi S., Lambani N., Kashyap P.L., Bisht D.S. (2017). Genetic engineering approaches to enhance oil content in oilseed crops. Plant Growth Regul..

[22] Mutanda T., Naidoo D., Bwapwa J.K., Anandraj A. (2020). Biotechnological Applications of Microalgal Oleaginous Compounds: current Trends on Microalgal Bioprocessing of Products. Front. Energy *Re*.

[23] Li R., Jin M., Du J., Li M., Chen S., Yang S. (2020). The magnesium concentration in yeast extracts is a major determinant affecting ethanol fermentation performance of zymomonas mobilis. Front. Bioeng. Biotechnol..

[24] Phukan M.M., Bora P., Gogoi K., Konwar B.K. (2019). Biodiesel from Saccharomyces cerevisiae: fuel property analysis and comparative economics. SN Appl. Sci..

[25] Yang Z., Ohlrogge J.B. (2009). Turnover of fatty acids during natural senescence of Arabidopsis, Brachypodium, and switch grass and in Arabidopsis β-oxidation mutants. Plant Physiol..

[26] Murashige T., Skoog F. (1962). A revised medium for rapid growth and bio assays with tobacco tissue cultures. Physiol. Plant..

[27] Holsters M., De Waele D., Depicker A., Messens E., Van Montagu M., Schell J. (1978). Transfection and transformation of Agrobacterium tumefaciens. MGG.

[28] Jefferson R.A., Kavanagh T.A., Bevan M.W. (1987). GUS fusions: beta-glucuronidase as a sensitive and versatile gene fusion marker in higher plants. EMBO J..

[29] Ahmed N., Nawaz S., Iqbal A., Mubin M., Butt A., Lightfoot D.A., Maekawa M. (2013). Extraction of high-quality intact DNA from okra leaves despite their high content of mucilaginous acidic polysaccharides. Bioscience Methods.

[30] Christie W.W. (2003).

[31] Browse J., McCourt P.J., Somerville C.R. (1986). Fatty acid composition of leaf lipids determined after combined digestion and fatty acid methyl ester formation from fresh tissue. Anal. Biochem..

[32] Wagner M., Hoppe K., Czabany T., Heilmann M., Daum G., Feussner I., Fulda M. (2010). Identification and characterization of an acyl-CoA: diacylglycerol acyltransferase 2 (DGAT2) gene from the microalga O. tauri. Plant Physiol. Biochem..

[33] Li Y., Beisson F., Pollard M., Ohlrogge J. (2006). Oil content of Arabidopsis seeds: the influence of seed anatomy, light and plant-to-plant variation. Phytochemistry.

[34] Chellamuthu M., Kumaresan K., Subramanian S., Muthumanickam H. (2019). Functional analysis of sesame diacylglycerol acyltransferase and phospholipid: diacylglycerol acyltransferase genes using in silico and in vitro approaches. Plant Mol. Biol. Rep..

[35] Pan X., Chen G., Kazachkov M., Greer M.S., Caldo K.M.P., Zou J., Weselake R.J. (2015). In vivo and in vitro evidence for biochemical coupling of reactions catalyzed by lysophosphatidylcholine acyltransferase and diacylglycerol acyltransferase. J. Biol. Chem..

[36] Chi X., Hu R., Zhang X., Chen M., Chen N., Pan L., Yu S. (2014). Cloning and functional analysis of three diacylglycerol acyltransferase genes from peanut (Arachis hypogaea L.). PLoS ONE.

[37] Liu X.Y., Ouyang L.L., Zhou Z.G. (2016). Phospholipid: diacylglycerol acyltransferase contributes to the conversion of membrane lipids into triacylglycerol in Myrmecia incisa during the nitrogen starvation stress. Sci. Rep..

[38] Yuan L., Mao X., Zhao K., Ji X., Ji C., Xue J., Li R (2017). Characterisation of phospholipid: diacylglycerol acyltransferases (PDATs) from Camelina sativa and their roles in stress responses. Biol. Open.

[39] Xue Y., Chen B., Win A.N., Fu C., Lian J., Liu X., Chai Y. (2018). Omega-3 fatty acid desaturase gene family from two ω-3 sources, Salvia hispanica and Perilla frutescens: cloning, characterization and expression. PLoS ONE.

[40] Schuhmann H., Lim D.K., Schenk P.M. (2012). Perspectives on metabolic engineering for increased lipid contents in microalgae. Biofuels.

[41] Scranton M.A., Ostrand J.T., Fields F.J., Mayfield S.P. (2015). Chlamydomonas as a model for biofuels and bio-products production. Plant J..

[42] Dyer J., Yurchenko O., Park S., Gidda S., Cai Y., Shockey J., Goodman J., Chapman K., Mullen R. (2015). Production of oil in plant vegetative tissues. FASEB J..

[43] Xu C., Shanklin J. (2016). Triacylglycerol metabolism, function, and accumulation in plant vegetative tissues. Annu. Rev. Plant Biol..

[44] Vanhercke T., El Tahchy A., Liu Q., Zhou X.R., Shrestha P., Divi U.K., Ral J.P., Mansour M.P., Nichols P.D., James C.N., Horn P.J (2014). Metabolic engineering of biomass for high energy density: oilseed-like triacylglycerol yields from plant leaves. Plant Biotechnol. J..

