# Supplementary material for: Modification of sesame (Sesamum indicum L.) for Triacylglycerol accumulation in plant biomass for biofuel applications
Source: Biotechnol Rep (Amst). 2021 Sep 11;32:e00668. doi: 10.1016/j.btre.2021.e00668 (PMC8449027; doi:10.1016/j.btre.2021.e00668)
Supplement: Supplementary file 1 [file mmc1.docx]

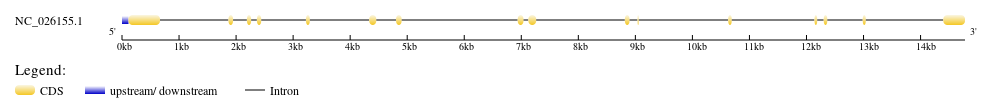


**DGAT1**


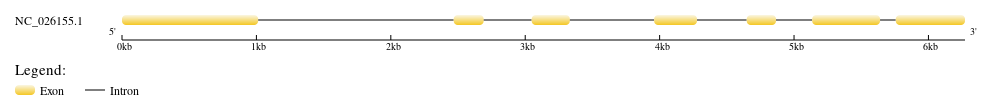


**PDAT1**


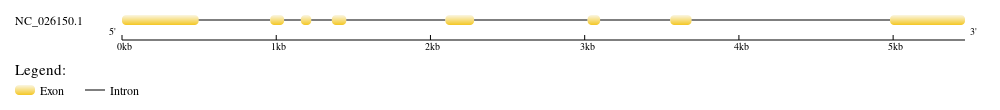


**FAD3**


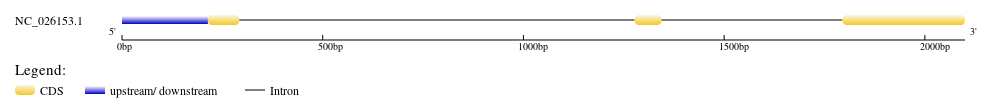


**Cyt b5 F**


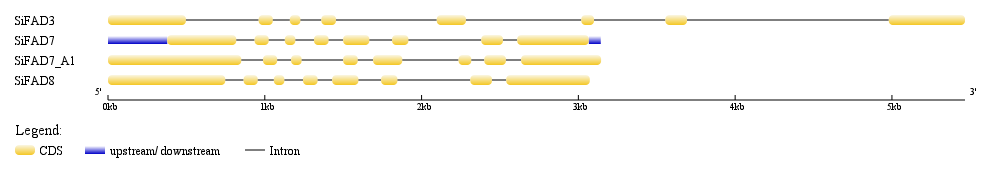


**Supplementary Figure 1: Gene structure of DGAT1, PDAT1, FAD3 and Cyt b5-F genes of Sesame**


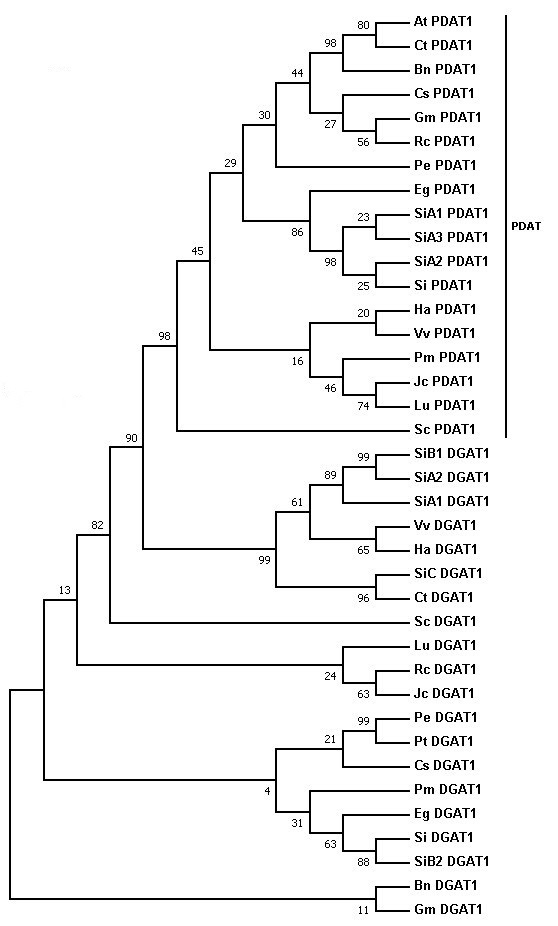


**PDAT1**

**DGAT1**

**Supplementary Figure 2:** Phylogenetic tree of DGAT1 and PDAT1 gene families reconstructed by the Maximum like hood (ML) method from complete alignment of 38 proteins sequences. Bootstrapping with 500 replicates was used to establish the confidence limits of the tree branches. Taxa terminologies are abbreviated using the first letter of the genus and the species name as given in the supplementary table.1

| **Species name** | **DGAT 1** | **PDAT 1** | |
| --- | --- | --- | --- |
| *Ricinus communis(Rc)* | NP_001310663.1 | NP_001310694.1 | |
| *Linum usitatissimum(Lu)* | AHA57450.1 | AHA57447.1 | |
| *Citrus sinensis(Cs)* | XP_006475486.1 | XP_006465948.1 | |
| *Camelina sativa(Ct)* | XP_010442900.1 | XP_010453452.1 | |
| *Brassica napus(Bn)* | NP_001303201.1 | CDX91106.1 | |
| *Arabidopsis thaliana(At)* | AAF19262.1 | NP_196868.1 | |
| *Glycine max(Gm)* | AAT73629.1 | XP_003548968.1 | |
| *Sesamum indicum(Si)* | NP_001291334.1  XP_011092556.1(A1)  XP_011088231.2(A2)  XP_011088232.2(B1)  XP_011075993.1(C)  XP_020553215.1(B2) | XP_020553631.1 XP_011093788.1(A1)  XP_011093787.1(A2)  XP_020553630.1(A3) | |
| *Populus euphratica(Pe)* | XP_011045601.1 | XP_011040053.1 | |
| *Helianthus annuus(Ha)* | OTF91364.1 | OTG03703.1 | |
| *Erythranthe guttata(Eg)* | XP_012849080.1 | XP_012851348.1 | |
| *Prunus mume(Pm)* | XP_008242379.1 | XP_008242198.1 | |
| *Jatropha curcas(Jc)* | ACA49853.1 | NP_001292930.1 | |
| *Saccharomyces cerevisiae(Sc)* | NP_014888.1 NP_014405.1 | | |
| *Vitis vinifera(Vv)* | XP_010647731.2 | | XP_002278397.1 |
| *Populus trichocarpa(Pt)* | XP_002330510.1 | | XP_002328081.1 |
| TOTAL | 20 | | 18 |

**Supplementary Table 1: The table shows the species names, gene names and accession numbers of DGAT1 and PDAT1 protein sequences used for the analysis.**


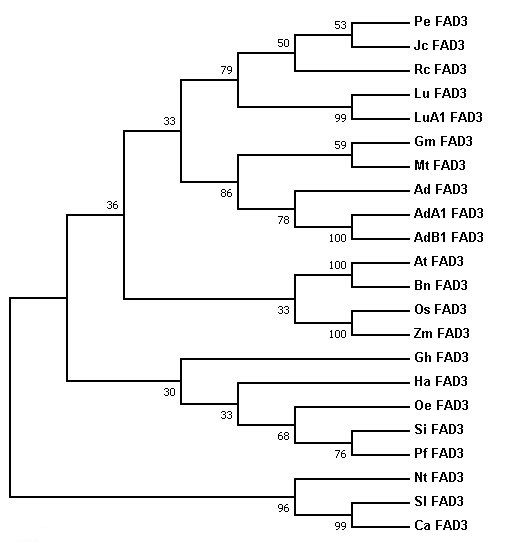


**Supplementary Figure 3:** Phylogenetic tree of FAD3 gene families reconstructed by the Maximum like hood (ML) method from complete alignment of 22 proteins sequences. Bootstrapping with 500 replicates was used to establish the confidence limits of the tree branches. Taxa terminologies are abbreviated using the first letter of the genus and the species name as given in the supplementary table.2

| **Species Name** | **FAD3** |
| --- | --- |
| *Sesamum indicum* (Si) | XP_011080789.1 |
| *Arabidopsis thaliana* (At) | NP_180559.1 |
| *Perilla frutescens* (Pf) | AAD15744.1 |
| *Linum usitatissimum* (Lu) | ABA02173.1  AFN53677.1(A1) |
| *Nicotiana tabacum* (Nt) | NP_001311727.1 |
| *Glycine max* (Gm) | NP_001237507.1 |
| *Populus euphratica* (Pe) | XP_011043784.1 |
| *Ricinus communis* (Rc) | XP_015578794.1 |
| *Helianthus annuus* (Ha) | XP_021976548.1 |
| *Olea europaea* (Oe) | AMJ42556.1 |
| *Gossypium hirsutum* (Gh) | NP_001313684.1 |
| *Solanum lycopersicum* (Sl) | NP_001233791.2 |
| *Jatropha curcas* (Jc) | XP_020532505.1 |
| *Capsicum annuum* (Ca) | XP_016575750.1 |
| *Medicago truncatula* (Mt) | XP_003615694.1 |
| *Arachis duranensis* (Ad) | XP_015967506.1  XP_015932035.1(A1)  XP_020983325.1(B1) |
| *Brassica napus* (Bn) | AAT09135 |
| *Oryza sativa* (Os) | BAA11397 |
| *Zea mays* (Zm) | NP_001149938 |
| Total | 22 |

**Supplementary Table 2: The table shows the species names, gene names and accession numbers of FAD3 protein sequences used for the analysis.**


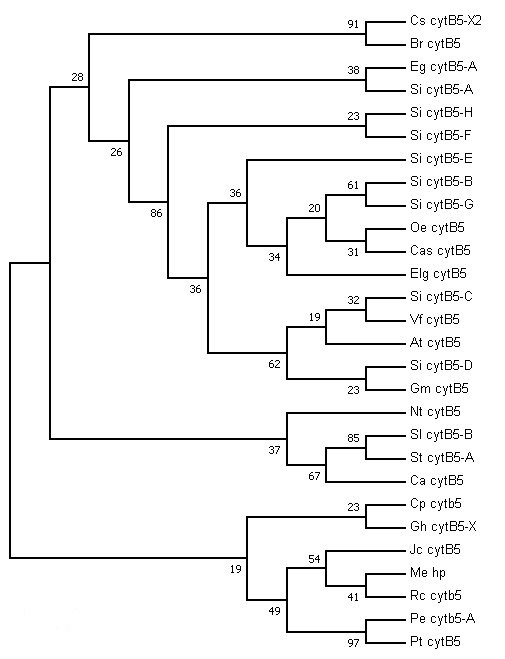


**Supplementary Figure 4:** Phylogenetic tree of cytochrome b5 gene families reconstructed by the Maximum like hood (ML) method from complete alignment of 28 proteins sequences. Bootstrapping with 500 replicates was used to establish the confidence limits of the tree branches. Taxa terminologies are abbreviated using the first letter of the genus and the species name as given in the supplementary table.3

| **Species Name** | **Cytochrome b5** |
| --- | --- |
| *Sesamum indicum (Si)* | XP_011070917.1(A)  XP_011089100.1(B)  XP_011075921.1(C)  XP_011093011.1(D)  XP_011091691.1(E)  XP_011089685.1(F)  XP_011089102.1(G)  XP_011078248.1(H) |
| *Erythranthe guttata (Eg)* | XP_012831698.1 |
| *Solanum lycopersicum(Sl)* | NP_001295135.1 |
| *Solanum tuberosum(St)* | XP_006348668.1 |
| *Capsicum annuum(Ca)* | XP_016547159.1 |
| *Gossypium hirsutum(Gh)* | XP_016667110.1 |
| *Jatropha curcas(Jc)* | XP_012067812.1 |
| *Nicotiana tabacum(Nt)* | XP_016443625.1 |
| *Manihot esculenta(Me)* | OAY59358.1 |
| *Ricinus communis(Rc)* | XP_002512059.2 |
| *Carica papaya(Cp)* | XP_021910387.1 |
| *Populus euphratica(Pe)* | XP_011011719.1 |
| *Populus trichocarpa(Pt)* | XP_002314997.3 |
| *Brassica rapa(Br)* | XP_009115356.1 |
| *Camelina sativa(Cs)* | XP_010460320.1 |
| *Olea europaea(Oe)* | XP_022887887.1 |
| *Camellia sinensis(Cas)* | XP_028126133.1 |
| *Elaeis guineensis(Elg)* | XP_010929918.1 |
| *Arabidopsis thaliana(At)* | AAL87348.1 |
| *Vernicia fordii(Vf)* | AAT84458.1 |
| *Glycine max(Gm)* | NP_001236501.2 |
| Total | 28 |

**Supplementary Table 3: The table shows the species names, gene names and accession numbers of cytochrome b5 protein sequences used for the analysis.**


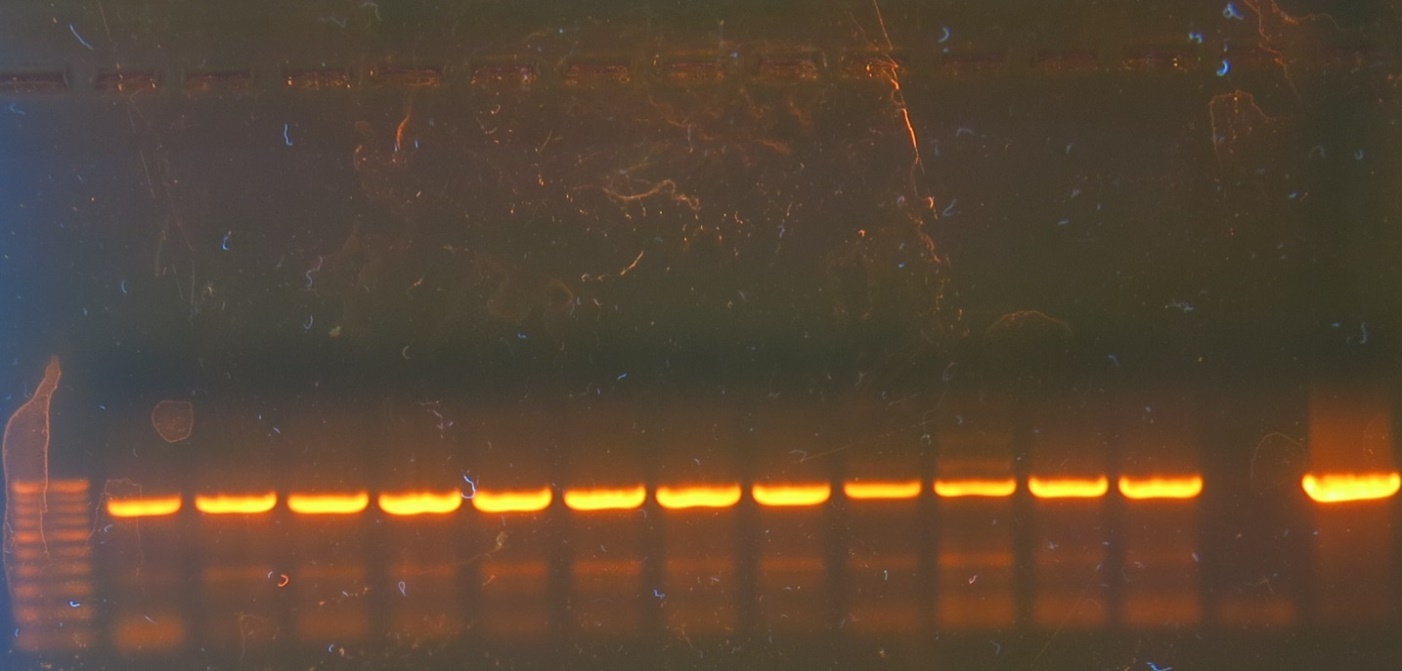


**Supplementary Figure 5: PCR amplification of NPTII using genomic DNA isolated from transgenic sesame lines**. M- 100bp ladder ,lanes 1-12 transformed sesame lines, lane 13- negative control (untransformed sesame plant ) and lane 14- positive control (pBI121 plasmid)

**M 1 2 3 4 5 6 7 8 9 10 11 12 13 14**

790bp

100bp

600bp


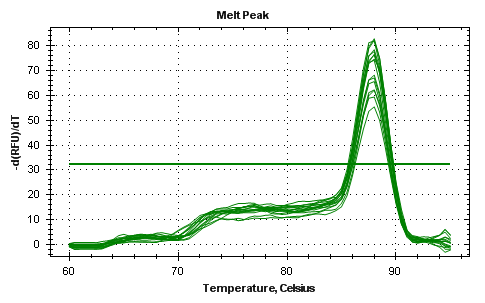


**Supplementary Figure 6: Melt curve analysis of SYBR qPCR assay for *NPTII* gene.** Melt curve analysis indicated that *NPTII* gene-specific qRT-PCR resulted in a single peak of amplification signal.
