# Supplementary material for: Modification of sesame (Sesamum indicum L.) for Triacylglycerol accumulation in plant biomass for biofuel applications
Source: Biotechnol Rep (Amst). 2021 Sep 11;32:e00668. doi: 10.1016/j.btre.2021.e00668 (PMC8449027; doi:10.1016/j.btre.2021.e00668)
Supplement: Supplementary file 2 [file mmc2.docx]

**RB**

**NOS Pro**

**NPT II**

**NOS Ter**

**CaMV 35S Pro**

**Xba I**

**DGAT1+FAD3**

**SnaBI**

**GUS**

**NOS Ter**

**LB**

**2.8Kb**

**A**

**RB**

**NOS Pro**

**NPT II**

**NOS Ter**

**CaMV 35S Pro**

**Xba I**

**PDAT1+FAD3**

**SnaBI**

**GUS**

**NOS Ter**

**LB**

**3.2Kb**

**B**

**RB**

**NOS Pro**

**NPT II**

**NOS Ter**

**CaMV 35S Pro**

**Xba I**

**Cyt b5-F+FAD3**

**SnaBI**

**GUS**

**NOS Ter**

**LB**

**1.6Kb**

**C**

**GUS**

**GUS**

**GUS**

**GUS**

**Supplementary** **Figure 4: A.** Schematic representation of binary vector pBI121 harboring DGAT1 and FAD3 under the control CaMV 35S promoter **B**. pBI121 harbors PDAT1 and FAD3 **C**.pBI121 harboring cytochrome b5-F and FAD3 gene.
