# Supplementary material for: Modification of sesame (Sesamum indicum L.) for Triacylglycerol accumulation in plant biomass for biofuel applications
Source: Biotechnol Rep (Amst). 2021 Sep 11;32:e00668. doi: 10.1016/j.btre.2021.e00668 (PMC8449027; doi:10.1016/j.btre.2021.e00668)
Supplement: Supplementary file 3 [file mmc3.docx]

**Supplementary Table 1: List of primers used for amplifying DGAT1, PDAT1, FAD3 and Cyt b5 genes**

| **Gene Name** | **Forward primer** | Reverse primer | **Gene ID** | **Linkage Group** |
| --- | --- | --- | --- | --- |
| DGAT1 | XbaI  5’-TAT**TCTAGA**ATGGCGATTTTGGACTC-3’ | XmaI  5´TAT**CCCGGG**CTACCTTGCACTAGCTTTTC-3´ | 105173637 | LG11 |
| PDAT1 | XbaI  5´-TAT**TCTAGA**ATGGCGATCATGAGGCGTAG -3´ | XmaI  5’TAT**CCCGGG**CTAAAGTCTTAATTTAATCCTTTCTG-3’ | 105173654 | LG11 |
| Cyt b5-F | XbaI  5’-TAT**TCTAGA**ATGGCTAAAGTTTTCACTTT-3’ | XmaI  5’-TAT**CCCGGG**TCATGCTGATGATTTGGAAA-3’ | [105163949](https://www.ncbi.nlm.nih.gov/gene/105163949) | LG6 |
| FAD3 | XmaI  5’-TAT**CCCGGG**ATGGCCGTAAT TTCAGGCC-3’ | SnaBI  5’TAT**TACGTA**ATAGTATTAGTTGGTATGGAGCTTTGA-3’ | [105170159](https://www.ncbi.nlm.nih.gov/gene/105170159) | LG9 |

**Supplementary Table 2: List of primers used for qRT-PCR analysis**

| **Gene Name** | **Forward primer** | **Reverse primer** |
| --- | --- | --- |
| FAD3 | 5’CGGAAGAGATCAATGGCGGTC -3’ | 5’- GATGTCCGCAATCCTGAACGG -3’ |
| Cyt b5-A | 5’-TCTTAAGGGCCTTCCACAGA-3’ | 5’-AGCCAACAACCACCGATAAC-3’ |
| Cyt b5-B | 5’-AGCAGCCTCAATACGACCAG-3’ | 5’-ACAGCCACACCCAAGATGAT-3’ |
| Cyt b5-C | 5’-CTCCACGACTTTGAGGAGGT-3’ | 5’-GACTTCATCACCCCCAGGAT-3’ |
| Cyt b5-D | 5’-GATGTTGGCCACAGTGCTAC-3’ | 5’-GAGGGGCAGATGCTGTCTTA-3’ |
| Cyt b5-E | 5’-CCCTGAAACGAGCTTACGTC-3’ | 5’-GCCAAACCCAAGATCAAGAG-3’ |
| Cyt b5-F | 5’-CCGGTGGTGATGATCTTCTG-3’ | 5’-AGAGCCTCTCCATCATGCTC-3’ |
| Ubiquitin | 5´-CACCAAGCCGAAGAAGATCAAG-3´ | 5´-CCTCAGCCTCTGCACCTTTC-3´ |
| QNPTII | 5’-GGATGATCTGGACGAAGAGC-3’ | 5’-CATGTGTCACGACGAGATCC-3’ |

**Supplementary Table 3: Basic parameters of FAD3 and Cyt b5 genes in *Sesamum indicum***

| **Gene name** | **mRNA accession** | **Protein accession** | **Protein length (aa)** | **Gene length** | **Molecular mass** | **pI** |
| --- | --- | --- | --- | --- | --- | --- |
| FAD3 | XM_011082487.1 | XP_011080789.1 | 409 | 1236bp | 46601.18 | 8.48 |
| Cyt b5-F | XM_011090800.1 | XP_011089102.1 | 149 | 450bp | 16617.94 | 5.27 |

**Supplementary Table 4: Predicted conserved domain and subcelluar localization of DGAT1, PDAT1, FAD3 and Cyt B5 genes of sesame**

| **Name** | **Conserved domain (SMART)** | | **Subcellular location** |
| --- | --- | --- | --- |
| DGAT1 | [Transmembrane region](javascript:domWin(2)) | 148-167,191-210,222-244,249-268,337-359,385-407,447-469,479-501,508-530 | Endoplasmic reticulum |
|  | [MBOAT](javascript:domWin(3)) | 179 to 533 |  |
| PDAT1 | [Transmembrane region](javascript:domWin(4)) | 70 to 92 | Chloroplast |
|  | [LCAT](javascript:domWin(5)) | 150 to 434 |  |
|  | [LCAT](javascript:domWin(10)) | 495 to 635 |  |
| FAD3 | [DUF3474](http://pfam.xfam.org/family?id=DUF3474) | 5 to 94 | Endoplasmic reticulum |
|  | [FA_desaturase](http://pfam.xfam.org/family?id=FA_desaturase) | 99 to 361 |  |
|  | Transmembrane region | 78-97,101-123,240-262 |  |
| Cyt b5-F | Heme/steroid binding domain | 5 to 78 | Endoplasmic reticulum |
|  | Transmembrane region | 123 to 145 |  |
